# Cool Headed Individuals Are Better Survivors: Non-Consumptive and Consumptive Effects of a Generalist Predator on a Sap Feeding Insect

**DOI:** 10.1371/journal.pone.0135954

**Published:** 2015-08-21

**Authors:** Orsolya Beleznai, Gergely Tholt, Zoltán Tóth, Vivien Horváth, Zsolt Marczali, Ferenc Samu

**Affiliations:** 1 Zoology Department, Plant Protection Institute, Centre for Agricultural Research, Hungarian Academy of Sciences, Budapest, Hungary; 2 Institute for Plant Protection, Georgikon Faculty, University of Pannonia, Keszthely, Hungary; 3 Eötvös Loránd University, Faculty of Science, Institute of Biology, Budapest, Hungary; 4 Lendület Evolutionary Ecology Research Group, Plant Protection Institute, Hungarian Academy of Sciences, Budapest, Hungary; 5 Institute of Genetics and Biotechnology, Faculty of Agricultural and Environmental Sciences, Szent István University, Gödöllő, Hungary; Hungarian Academy of Sciences, HUNGARY

## Abstract

Non-consumptive effects (NCEs) of predators are part of the complex interactions among insect natural enemies and prey. NCEs have been shown to significantly affect prey foraging and feeding. Leafhopper's (Auchenorrhyncha) lengthy phloem feeding bouts may play a role in pathogen transmission in vector species and also exposes them to predation risk. However, NCEs on leafhoppers have been scarcely studied, and we lack basic information about how anti-predator behaviour influences foraging and feeding in these species. Here we report a study on non-consumptive and consumptive predator-prey interactions in a naturally co-occurring spider–leafhopper system. In mesocosm arenas we studied movement patterns during foraging and feeding of the leafhopper *Psammotettix alienus* in the presence of the spider predator *Tibellus oblongus*. Leafhoppers delayed feeding and fed much less often when the spider was present. Foraging movement pattern changed under predation risk: movements became more frequent and brief. There was considerable individual variation in foraging movement activity. Those individuals that increased movement activity in the presence of predators exposed themselves to higher predation risk. However, surviving individuals exhibited a ‘cool headed’ reaction to spider presence by moving less than leafhoppers in control trials. No leafhoppers were preyed upon while feeding. We consider delayed feeding as a “paradoxical” antipredator tactic, since it is not necessarily an optimal strategy against a sit-and-wait generalist predator.

## Introduction

The consumption of prey by a predator is one of the most important ecological interactions, but costly anti-predator behaviour of prey, called non-consumptive effects (NCEs), also have considerable impact on ecological systems [1, 2]. The wide ranging behavioural responses of prey to the presence or cues of predators may result in reduced feeding [3], a lower quality diet [4, 5], physiological stress [6] or may be detrimental to reproduction [7]. A meta-analysis of studies in terrestrial systems showed substantial and significant decrease in foraging effort as a consequence of increased predation risk [8]. Such negative impacts of NCEs have the potential to cascade through ecological systems, affecting productivity and diversity at lower trophic levels [1].

Under predation risk, prey animals face a problem of optimization: they have to trade off foraging, feeding and reproduction against anti-predator vigilance, refuge seeking or fleeing [9, 10]. While a number of experimental results demonstrate that reduced activity enhances survival of the prey [11, 12], there are contrasting models which suggest increased activity resulting in higher elusiveness might also be a good tactic to avoid predation [13]. More recently, as variation in anti-predator behaviour at the level of the individual is gaining attention, it has been documented that responses to predator encounters interact with behavioural syndromes [14]. Sap feeding hemipteran insects are especially vulnerable to predation when their stylet is inserted in the plant tissues. Consequently, their decision whether to feed or for how long to feed should markedly depend on predation risk.

Here we report a study on the behavioural aspects of non-consumptive predator-prey interactions and possible consumptive outcome in a leafhopper-spider system. The grass-feeding leafhopper *Psammotettix alienus* (Dahlbom) (Hemiptera: Cicadellidae) is an important pest of cereals, and the only known vector of Wheat Dwarf Virus (WDV, Geminiviridae) [15], which causes serious damage to cereal crops. *P*. *alienus* has a lengthy phloem feeding phase [16], during which leafhoppers are essentially anchored to the plant by the opened serrate tip of their stylet [17], which–even if a potential predator is detected–can only be removed after several seconds. Virus acquisition and inoculation takes place during phloem feeding [18, 19]. Thus, the length of phloem feeding can be critical both for predator avoidance and for the effectiveness of virus transmission.

In NCE studies, spiders are frequently used as model predators because they are handled and manipulated easily [20], leave chemical cues in form of silk [3], and their NCEs often cascade through several trophic levels [21]. In this study we used a natural enemy of the leafhopper, the common agrobiont spider *Tibellus oblongus* (Walckenaer) (Araneae: Philodromidae) [22]. *T*. *oblongus* is a generalist sit-and-wait predator, active mostly during daytime. Without a web, it ambush hunts for a wide variety of prey [23–25]. The species mostly occurs in the foliage of grassy vegetation [26], perching on grass or cereal leaves, precisely in the microhabitat where *P*. *alienus* feeds.

In order to understand the complex interactions among predators and sap feeding vector herbivores, it is first critical to document how predators impact herbivores. Focusing on NCEs, we hypothesized that the leafhoppers would alter their foraging activity and feeding behaviour when exposed to predation risk. We predicted that, compared to a control situation, under predation risk foraging will be reduced, which can be detected by change in the movement pattern, and we also expected that phloem feeding, detected by the production of honeydew, will be reduced by delaying and/or shortening of feeding events. We were also interested in individual variation in anti-predator behaviour and its bearing on prey survival. We predicted that under identical predation risk the individual with lower foraging movement and feeding activity will have a higher probability of survival. To test this, we exposed two leafhoppers to one spider, and compared foraging movement activity and feeding rate of victims and survivors facing the same spider.

## Materials and Methods

### Experimental animals

#### Collection and keeping

To initiate laboratory populations, *T*. *oblongus* and *P*. *alienus were co*llect*ed from fa*llow and grassland areas near Budapest and Székesfehérvár, Hungary (47°32'58.94"N, 18°55'48.81"E; 47°14'40.73"N, 18°25'40.45"E, respectively) by sweep net and suction sampling. After collection, *P*. *alienus* were kept *in a climat*e room (L/D: 16/8 h, 23/16°C regime) on potted young barley (*Hordeum vulgare* L. cv M*V Jubilant); po*ts caged with fine meshing. Juveniles were kept until adulthood; only adults were used for the experiments. Animals, randomly selected for the trials, were withheld from food for 30 min and their sex was determined. Spider individuals were kept under the same conditions, in individual plastic vials with moistened Plaster of Paris base to maintain humidity, fed *ad libitum* with *Drosophila melanogaster (Meigen)* t*wice a week*. For the predator treatment we selected juvenile spiders randomly from the laboratory stock. Small spiders were not selected; the body mass range was 5–15 mg. Prior to the experiment, spiders received no food for one week to standardize hunger level. Their body mass was measured to the nearest 0.1 mg before the trials. Individual spiders and leafhoppers were only used once in our experiments.

#### Ethics statement

None of the arthropod species used in the present experiment are protected in Hungary. Under Hungarian law (act 348/2006, paragraph 10 (3)), unprotected arthropods can be freely collected in fields and their use in laboratory experiments requires no permit or approval by ethics committee. Collecting sites (crop fields and grassland patches) were either owned by the research institute (Plant Protection Institute, Centre for Agricultural Research, Hungarian Academy of Sciences, Budapest) or were publicly accessible, therefore no permit was needed to access them. During experimentation we avoided causing any unnecessary harm, suffering or distress to the study subjects.

### Behavioural observations

#### Procedures of observations

Observational trials of *P*. *alienus* were conducted i*n mesocosm* arenas in the presence and absence of the spider. Each arena consisted of a pot (10 cm diam.) pre-planted with 3 stems of barley in sterilised soil. Pots were fitted with plexiglass cylinders (6 cm diameter, 20 cm high), covered with fine meshing. Arenas received uniform lighting, temperature was 23°C, relative humidity 30–50%. Trials were performed when plants were at 2–3 leaves stage.

During a trial, after placing the animals into the arena, their behaviour was monitored visually for the prescribed period of the trial. We used the Observer XT software (version 11, Noldus Information Technology, Wageningen, Netherlands) to record the timing and duration of behavioural events.

#### Movement activity observations

We made observations of movement activity in 29–42 min trials (Table 1). These were run in two series, in August-November 2012 and in October-November 2013. We recorded the following behavioural events: (i) the occurrence of predation; (ii) moving events, observed separately both for spiders and leafhoppers, which were more or less continuous change of position by walking, or repositioning by jumping; (iii) stationary events, being mutually exclusive to moving events; (iv) honeydew production by the leafhopper; (v) feeding by the spider. If a behavioural event ended artificially (the leafhopper was preyed on or observation ended), then we considered the last behaviour as incomplete (censored). Such incomplete behaviours were included in count type variables (they were counted), but their durations were not taken into account in statistical tests.

**Table 1:** Subjects present in observational trials.

		Trials					
Observation	Leafhopper number	Spider treatment		Leafhopper control		Spider control	
**Feeding (3 h)**	2L	L L S	(N = 44)	L L	(N = 44)		
**Movement activity (c. 30 min)**	1L	L S	(N = 96)	L	(N = 96)	S	(N = 96)
	2L	L L S	(N = 100)	L L	(N = 100)	S	(N = 100)

L = leafhopper, S = spider, in brackets the number of trials run.

In movement activity observations we had three trials (arenas) per session: a spider treatment, a leafhopper control and a spider control (Table 1). We ran two setup variants, one with one leafhopper per trial (1L), and another with two leafhoppers (2L).We refer to this as ‘leafhopper number’ variable. In the case of 2L setups, one of the individuals was marked with a small dot of silver glue, the same applied in electrical penetration graph studies [16]. We found no effect of this marking on the probability of becoming a survivor or victim during the trials (test of independence; *χ* ^2^_1_ = 0.15, *P* = 0.695). The main reason for having 2L situations was that we could compare the behaviour of prospective victim and survivor individuals and could follow the behaviour of a survivor leafhopper after the spider attacked the other individual.

**Fig 1:**
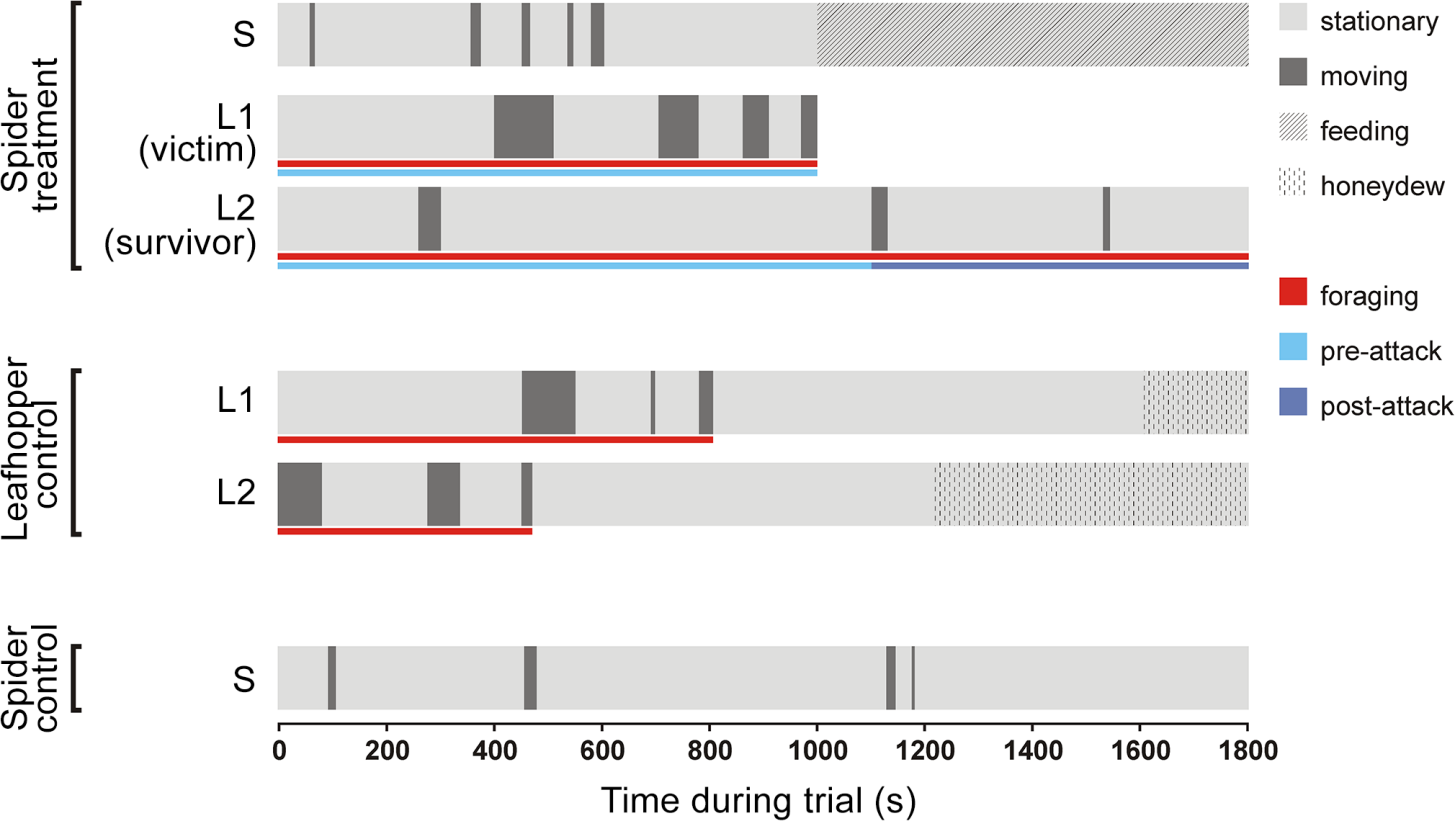
Schematic representation of the movement activity observations in a two-leafhopper session. Activity pattern and feeding events are depicted for subjects in the trials, foraging, pre-attack and post-attack periods are additionally marked. In two leafhopper trials, when the victim was attacked, if the current behaviour of the survivor extended beyond the attack on the victim, then this extension was included in the pre-attack period.

The sequence of moving and stationary events (pattern of movement activity) was compared in various time windows, or “periods”, dictated by the research question (Fig 1). Questions regarding movement activities during foraging were studied in the ‘foraging period’, that is the period before a leafhopper started to feed. Honeydew production always occurred in the course of a stationary event. Since a variable but often lengthy period is needed for the leafhoppers to reach the phloem with their stylets [16], we regarded the whole stationary event during which honeydew was produced as part of the feeding process, and therefore excluded from the foraging period (Fig 1). If the leafhopper did not feed, then all events of the trial were counted as foraging period.

Questions regarding the effect of predation were studied during ‘pre-attack’ and ‘post-attack periods’ comprising two levels of the variable ‘predation period’ (Fig 1). Victim behaviour could only be studied during the pre-attack period, but survivor behaviour could be compared between pre- and post-attack periods. During post-attack periods spiders were effectively feeding on their prey for the whole, or nearly the whole period (spider feeding events did not end until the end of observation in 113 cases out of the total 127; the mean duration of uncensored feeding events was 18.1 min). We compared trials where predation occurred (N = 53) to trials where spider was present but no predation occurred (N = 47). We have artificially created predation periods also in the no predation trials by randomly assigning one of the attack times of the predation trials to no predation trials. We thereby created artificial pre- and post-attack periods with nearly identical duration distributions in both predation and no predation trials.

#### Feeding observations

Feeding observations were carried out in 3 h trials and focused on the duration of *P*. *alienus* feeding; m*ovement ac*tivity was not recorded here. These longer observations were needed because often both the initiation of feeding and the total feeding time were longer than the duration of the movement activity observations. We only ran 2L sessions (Table 1). Since spider behaviour (apart from predation) was not observed, we had no spider control trials. There was one series of the observation sessions in April 2013.

### Statistical analysis

#### Statistical procedures

Response variables, random terms, fixed predictors and observed variables of subject states are described below, and listed in detail in Table 2. We applied linear mixed-effect models (LME) to analyse the effect of predators on various aspects of foraging movements using the ‘nlme’ package of R 3.1.0 [27, 28]. Subjects and experimental units were used as random blocking factors where applicable. The necessity of each random factor was investigated by comparing the models with and without the given factor using likelihood ratio tests. When random effects were found negligible, LME models shrank to linear models (LM). In all model fittings we used backward removal procedure, starting with the full models containing all explanatory variables (main effects and specific interactions), then dropped the predictor with the highest P-value in each step until only P ≤ 0.05 effects remaine*d* (if there were any) in the final models [29]. AIC values and Akaike weights of the final and other candidate models are presented in S1 Table. Requirements of the fitted models were checked by plot diagnosis. For significant predictors of the final models we estimated effect sizes [30] as the proportion of variance explained by each trait (*η* ^2^ and its 95% CI) [31]. Parameter estimates of the predictors in the final models together with their standard error are shown in S2 Table.

**Table 2:** Behavioural responses (D) and potential predictors (I) used in the fitted models as dependent and independent variables, and observed variables (O) used in proportions and Chi square tests.

Variable name	Description	Role / Type^1^
Number of movements	number of moving events	D / count
Duration of moving events	duration of moving events	D / cont
Duration of stationary events	duration of stationary events	D / cont
Duration of feeding	duration of leafhopper feeding events	D / cont
Moving % foraging period	total movement duration as % of foraging period	D / cont
Time	starting time of an event (relative to start of trial)	I / cont
Time to feed	starting time of the first feeding event in a trial	I / cont
Subject	observed individual	I / rnd block
Trial	observations of one arena	I / rnd block
Session	trials observed in parallel	I / rnd block
Series	sessions run in the same period of year	I / rnd block
Spider	presence of spider in a trial (present/control)	I / nominal
Predation	whether predation occurred in a trial (yes/no)	I / nominal
Prey	whether the individual became prey (yes/no)	I / nominal
Status	leafhopper status (control/victim/survivor)	I / nominal
Sex	leafhopper sex (male/female)	I / nominal
Movement state	prey movement state (at time of predation)	O / nominal
Leafhopper number	number of *Psammotettix* individuals in a trial (1L/2L)	I / nominal
Feeding status	whether a leafhopper started to feed (produce honeydew) during a trial (fed/not fed)	O / nominal
Predation period	period within a trial (pre-attack/post-attack)	I / nominal
Observation duration	duration of an observed period or a whole trial	I / cont
Spider weight	weight (mg) of *Tibellus* individuals	I / cont

^1^ cont = continuous, rnd = random

It can be assumed that a leafhopper placed on a host plant will eventually start to feed; likewise, when feeding started it will inevitably end at some time point. Therefore we analysed time to the first feeding (‘time to feeding’) and ‘duration of feeding’ events on a survival analysis platform in JMP 6.0 [32] using the feeding observation data-set. We applied a Cox Proportional-hazards (CPH) model [33], used effectively for event–time data in foraging studies [34]. If feeding did not start or end until the end of an observation or a predatory event, those cases were marked as censored.

We applied proportion tests for the investigation of the equality of proportions (case of moving/stationary states of prey and non-prey leafhoppers at the moment of predation), and Chi square tests to test for independence of frequency data (number of individuals feeding, not feeding, becoming victims or survivor). Variables were subjected to logarithm (duration variables) or square-root transformation (movement number variable) to improve their fit to normal distribution. All tests were two-tailed with *α* = 0.05. We numbered each statistical model, listed in Table 3 and described briefly below. Models are grouped by basic questions they try to answer.

**Table 3:** The effect of different variables in statistical models and tests on leafhopper foraging movement activity and feeding.

{Model No.} Dependent variable (Observation^a^)	Predictors	df	Test statistics F/χ^2^	*η* ^2^ (95% CI)	*P*
**Q1** ^**d**^ **: Does spider presence affect leafhoppers’ activity?**					
**{1}** Duration of moving events^b^ (M)	**Observation duration** ^**b**^	**1,442**	**12.70**	**0.03 [0.01–0.07]**	**0.0004**
	**Time** ^**b**^	**1,1339**	**27.34**	**0.02 [0.01–0.04]**	**<0.0001**
	**Sex**	**1,442**	**1.26**	**0 [0–0.02]**	**0.263**
	**Spider**	**1,442**	**16.33**	**0.04 [0.01–0.08]**	**<0.0001**
	**Spider × Sex**	**1,442**	**7.47**	**0.02 [0–0.05]**	**0.007**
	Leafhopper number	1,441	0.08	-	0.784
	Spider × Time^b^	1,1338	0.06	-	0.808
**{2}** Duration of stationary events^b^ (M)	**Observation duration** ^**b**^	**1,445**	**91.86**	**0.17 [0.11–0.23]**	**<0.0001**
	**Time** ^**b**^	**1,1481**	**87.20**	**0.06 [0.04–0.08]**	**<0.0001**
	**Spider**	**1,445**	**31.69**	**0.07 [0.03–0.12]**	**<0.0001**
	**Leafhopper number**	**1,445**	**10.00**	**0.02 [0–0.06]**	**0.002**
	Sex	1,444	0.16	-	0.692
	Spider × Sex	1,443	0.09	-	0.766
	Spider × Time^b^	1,1480	0.92	-	0.337
**{3}** Number of movements^c^ (M)	**Observation duration**	**1,128**	**245.59**	**0.66 [0.56–0.72]**	**<0.0001**
	**Spider**	**1,326**	**10.09**	**0.03 [0.004–0.07]**	**0.002**
	**Leafhopper number**	**1,326**	**5.14**	**0.02 [0–0.05]**	**0.024**
	Sex	1,127	1.53	-	0.218
	Spider × Sex	1,126	2.16	-	0.145
**{4}** Moving % foraging period^b^ (M)	**Observation duration** ^**b**^	**1,123**	**33.56**	**0.21 [0.10–0.33]**	**<0.0001**
	Spider	1,321	1.31	-	0.254
	Leafhopper number	1,321	2.32	-	0.128
	Sex	1,122	2.71	-	0.103
	Spider × Sex	1,121	0.71	-	0.403
**Q2: Are nearby leafhoppers’ activity affected by act of predation and consumption?**					
**{5}** Number of movements^c^ (M)	**Observation duration** ^**b**^	**1,196**	**86.74**	**0.31 (0.20–0.40)**	**<0.0001**
	**Predation period**	**1,196**	**6.19**	**0.03 (0.001–0.09)**	**0.014**
	Predation	1,195	0.06	-	0.815
	Sex	1,195	0.35	-	0.553
	Predation period × Predation	1,194	1.68	-	0.196
**{6}** Duration of moving events^b^ (M)	**Sex**	**1,127**	**13.14**	**0.09 (0.02–0.20)**	**0.0004**
	Predation period	1,126	3.25	-	0.074
	Observation duration^b^	1,126	0.45	-	0.504
	Predation	1,126	0.02	-	0.902
	Predation period × Predation	1,124	0.01	-	0.941
**Q3: Is leafhopper feeding affected by spider presence?**					
**{7}** Time to feed (F)	**Spider**	**1**	**13.68**	**-**	**0.0002**
	**Sex**	**1**	**12.03**	**-**	**0.0005**
	**Spider × Sex**	**1**	**4.8**	**-**	**0.028**
**{8}** Duration of feeding (F)	Spider	1	1.55	-	0.212
**{9}** Feeding status/ Spider (F)	-	**1**	**27.18**	**-**	**<0.0001**
**{10}** Feeding status/ Spider (M)	-	**1**	**61.57**	**-**	**<0.0001**
**{11}** Feeding status/ Prey (F)	-	**1**	**6.43**	**-**	**0.011**
**{12}** Feeding status/ Prey (M)	-	1	1.57	-	0.209
**Q4: Is leafhopper activity a predictor of becoming victim?**					
**{13}** Number of movements^c^ (M)	**Observation duration** ^**b**^	**1,138**	**51.31**	**0.27 [0.15–0.38]**	**<0.0001**
	**Prey**	**1,138**	**29.94**	**0.18 [0.08–0.29]**	**<0.0001**
	Sex	1,137	0.27		0.601
	Sex × Prey	1,136	0.03	-	0.858
**{14}** Duration of moving events^b^ (M)	**Observation duration** ^**b**^	**1,68**	**8.27**	**0.11 [0.01–0.25]**	**0.005**
	Sex	1,34	0.21	-	0.646
	Prey	1,34	0.34	-	0.561
	Sex × Prey	1,32	1.57	-	0.220
**{15}** Moving % foraging period^b^ (M)	**Observation duration** ^**b**^	**1,261**	**35.84**	**0.12 [0.06–0.20]**	**<0.0001**
	**Status**	**2,261**	**3.74**	**0.03 [0–0.07]**	**0.025**
	Sex	1,260	3.25	-	0.073
	Sex × Status	2,258	0.49	-	0.613
**{16}** Movement state/Prey (M)	**-**	**1**	**61.07**	**-**	**<0.0001**
**Q5: Is leafhopper activity correlated with spider activity?**					
**{17}** Number of movements^c^ (M)	**Observation duration** ^**b**^	**1,265**	**72.47**	**0.21 [0.13–0.30]**	**<0.0001**
	**Leafhopper number**	**1,265**	**27.55**	**0.09 [0.04–0.16]**	**<0.0001**
	**Prey**	**1,265**	**38.62**	**0.13 [0.06–0.20]**	**<0.0001**
	**Spider activity**	**1,265**	**3.91**	**0.01 [0–0.06]**	**0.049**
	Spider weight	1,264	3.19	-	0.075
	Prey × Spider activity	1,264	0.13	-	0.715
**{18}** Duration of moving events^b^ (M)	**Observation duration** ^**b**^	**1,167**	**5.53**	**0.03 [0.001–0.10]**	**0.020**
	**Spider activity**	**1,167**	**7.32**	**0.04 [0.003–0.11]**	**0.008**
	Leafhopper number	1,166	0.08	-	0.774
	Prey	1,50	1.40	-	0.242
	Spider weight	1,166	0.63	-	0.428
	Prey × Spider activity	1,49	2.23	-	0.142

Test statistics and *P*-values for the non-significant predictors were obtained by including them one by one into the final model (in bold). Test statistics is *F* for all LME/LM models {1–6}, {13–15}, {17–18} and *χ* ^2^ for CPH models {7–8}, Chi square test {9–12} and the proportion test {16}.

^a^Observations: M = Movement activity observation, F = Feeding observation

^b^log-transformed

^c^square-root transformed

^d^numbered question referring to questions in Material and Methods

#### Statistical models and tests of predation risk effects

Question 1: Does spider presence affect leafhoppers’ activity? The effect of predation risk (variable ‘spider’; presence of spider levels: present/control) on the movement activity pattern of leafhoppers during the foraging period was modelled for the duration of moving events {1}, the duration of stationary events {2}, the number of movements {3} and the percent time leafhoppers spent moving during the foraging period {4} (Table 3).

Question 2: When predation and ensuing consumption of prey happens, are the activities of a nearby leafhopper affected? The effect of actual predation (variable ‘predation’; levels: predation yes/no) on survivors was tested in 2L trials by comparing movement activity pattern between the two levels (pre- / post-attack periods) of ‘predation period’. Since predation periods were defined for no predation trials as well (see Movement activity observations section), we established a two-way arrangement, where the effect of ‘predation period’ and ‘predation’ was analysed by LME models (Table 3) on the number of movements {5} and the average duration of moving events {6} of the leafhoppers.

Question 3: Is leafhopper feeding affected by spider presence? We modelled time to feeding and duration of feeding events by CPH models on data from the 3 h long feeding observations. Spider and sex were included in time to feeding model {7}, whereas sex was not considered in feeding duration model {8} because in spider treatment trials only 2 female and 8 male leafhoppers started feeding, compared to 28 female and 22 male individuals feeding in control trials. Separately for both the feeding observation and the movement activity observation datasets, we also tested for independence between leafhopper feeding status (fed/not fed) and spider presence (present/control) {9,10}, and leafhopper feeding status and prey status (victim/survivor) {11,12}.

Question 4: Is leafhopper activity a predictor of becoming victim? We examined which movement activity pattern proved better at avoiding predation and which made the leafhopper more prone to it, by analysing the potential behavioural differences between victim and survivor leafhoppers. In one approach, considering the pre-attack period, we compared number of movements {13} and the average duration of moving events {14} between survivors and victims (control leafhoppers excluded). Then, considering the respective foraging periods, we compared the percent time spent moving during foraging among leafhoppers of control, survivor and victim status {15}. In another approach we analysed whether moving or not moving (levels of the variable ‘movement state’) was the more likely behaviour occurring at the time of the predation, analysed by a proportion test {16}.

Question 5: Is leafhopper activity correlated with spider activity? The potential relationship between the activity of spiders and leafhoppers within the same trial was analysed both for the number of movements {17} and for the average duration of moving events {18} of leafhoppers during the pre-attack period (Table 3). Since movement number and movement durations (log-transformed to improve its fit to normal distribution) of the spiders were highly correlated, we performed principal component analyses (PCA) on these two variables and considered the first principal component as a proxy for spider activity in models {17–18}. The first component (spider activity, henceforward) was positively correlated with both movement measures and accounted for 66% of the total variance.

## Results

### Effect of perceived risk on movement patterns

**Fig 2:**
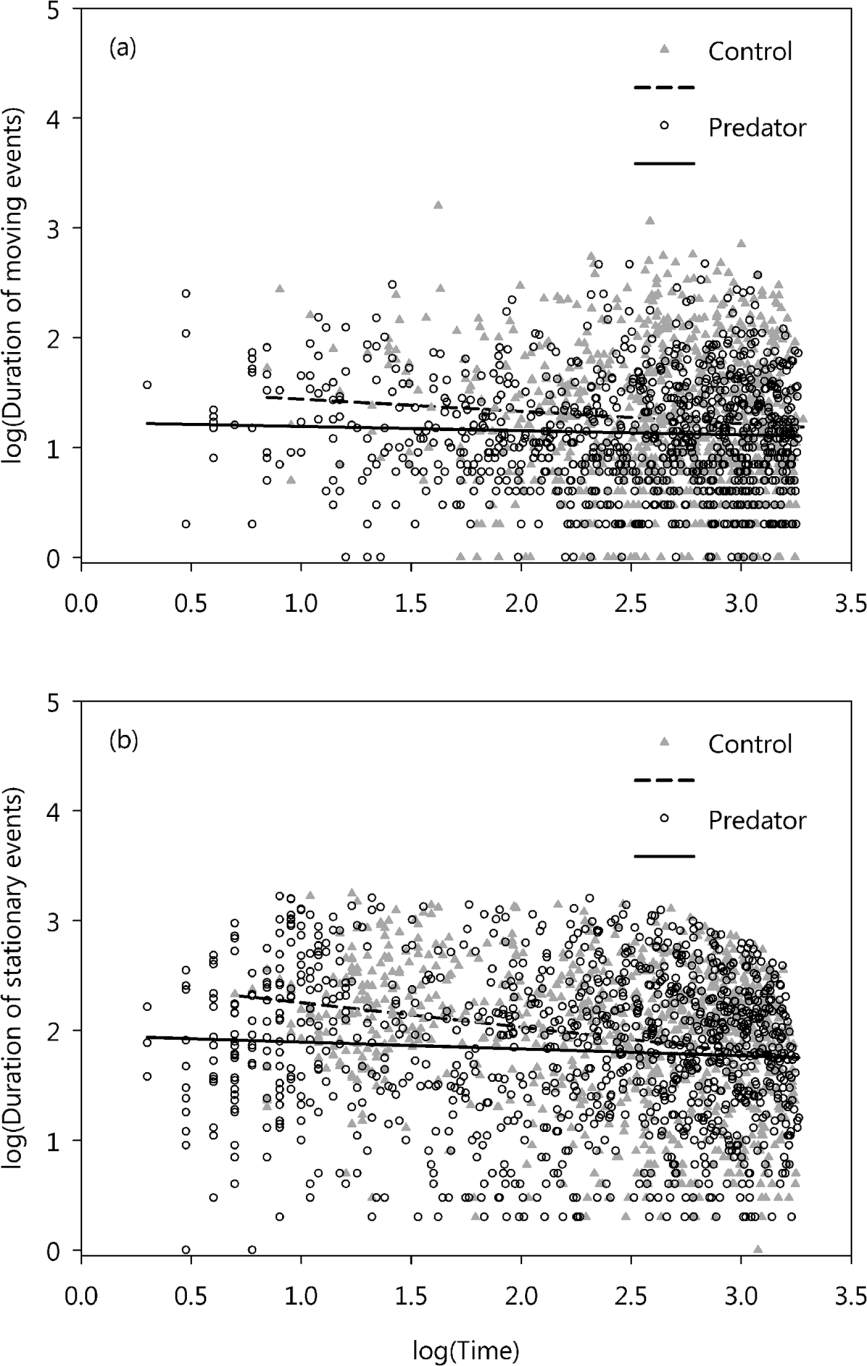
Change in moving and stationary event durations as a function of time. Relationship between the start of event and event duration (both log-transformed) during moving (a) and being stationary (b) in the foraging period.

Question 1: Leafhoppers changed their pattern of movement activity during foraging if they were exposed to predation risk. We found that the average duration of both moving and stationary events became slightly, but significantly shorter if a spider was present (Fig 2, Table 3 {1,2}). The shortening of all event types should result in more events, and indeed there were significantly more movement events in spider treatments (Table 3 {3}). However, shorter but more numerous events did not change the overall duration leafhoppers spent moving in the presence of a spider (Table 3 {4}).

We also observed a “background” shortening of the duration of moving and stationary events over the course of the trials that happened independently of spider presence (Fig 2). The duration of both moving and stationary events weakly but significantly decreased with time (Table 3 {1,2}). Also, the duration of moving events was longer in males compared to females when a spider was present (Table 3 {1}), and the duration of stationary events was longer where there were two leafhoppers present (Table 3 {2}). In all instances, Spider × Time interactions were not significant (Table 3 {1,2}), thus the slope of the “background” temporal changes in event durations was independent of spider presence, as the temporal trends found were similar between spider and control trials.

**Fig 3:**
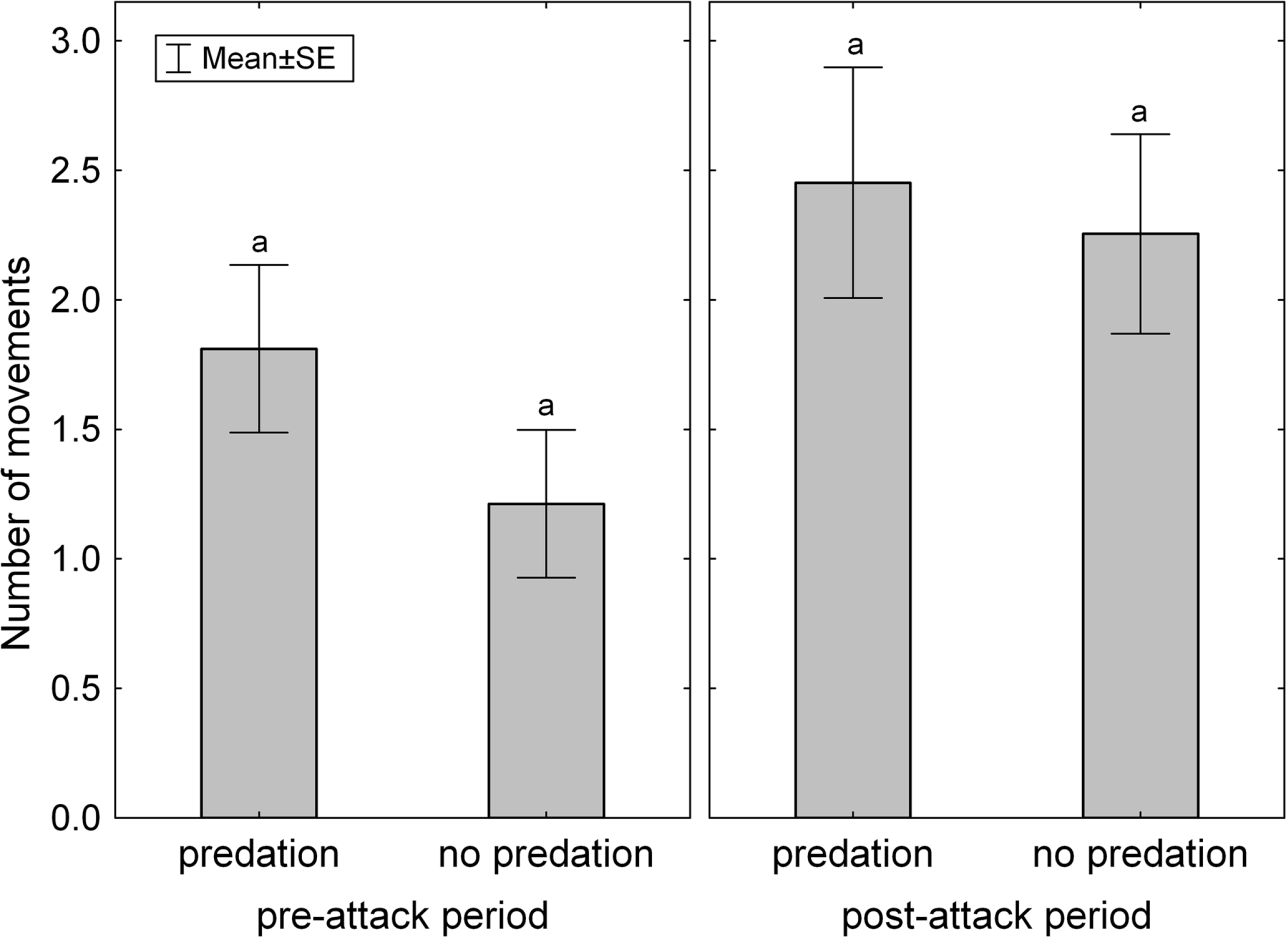
Number of movement events in survivor leafhoppers in trials with or without predation during the pre-attack and post-attack periods. The same letters signify that none of the groups were not significantly different (at P = 0.05) according to a Tukey HSD test.

Question 2: We found that in 2L trials where one leafhopper fell as victim, the survivor individual moved more frequently in the post-attack period (during which the victim individual was fed on by the spider) than in the pre-attack period. However, if we designate artificial pre- and post-attack periods to trials where spider was present but no predation occurred, then leafhoppers compared within the same periods showed no difference either in the number of movements (Fig 3, Table 3 {5}) or in the duration of moving events (Table 3 {6}). Thus, predation on the victim individual did not exert a significant effect on movement activity pattern of survivor individuals.

### Effect of risk on leafhopper feeding

**Fig 4:**
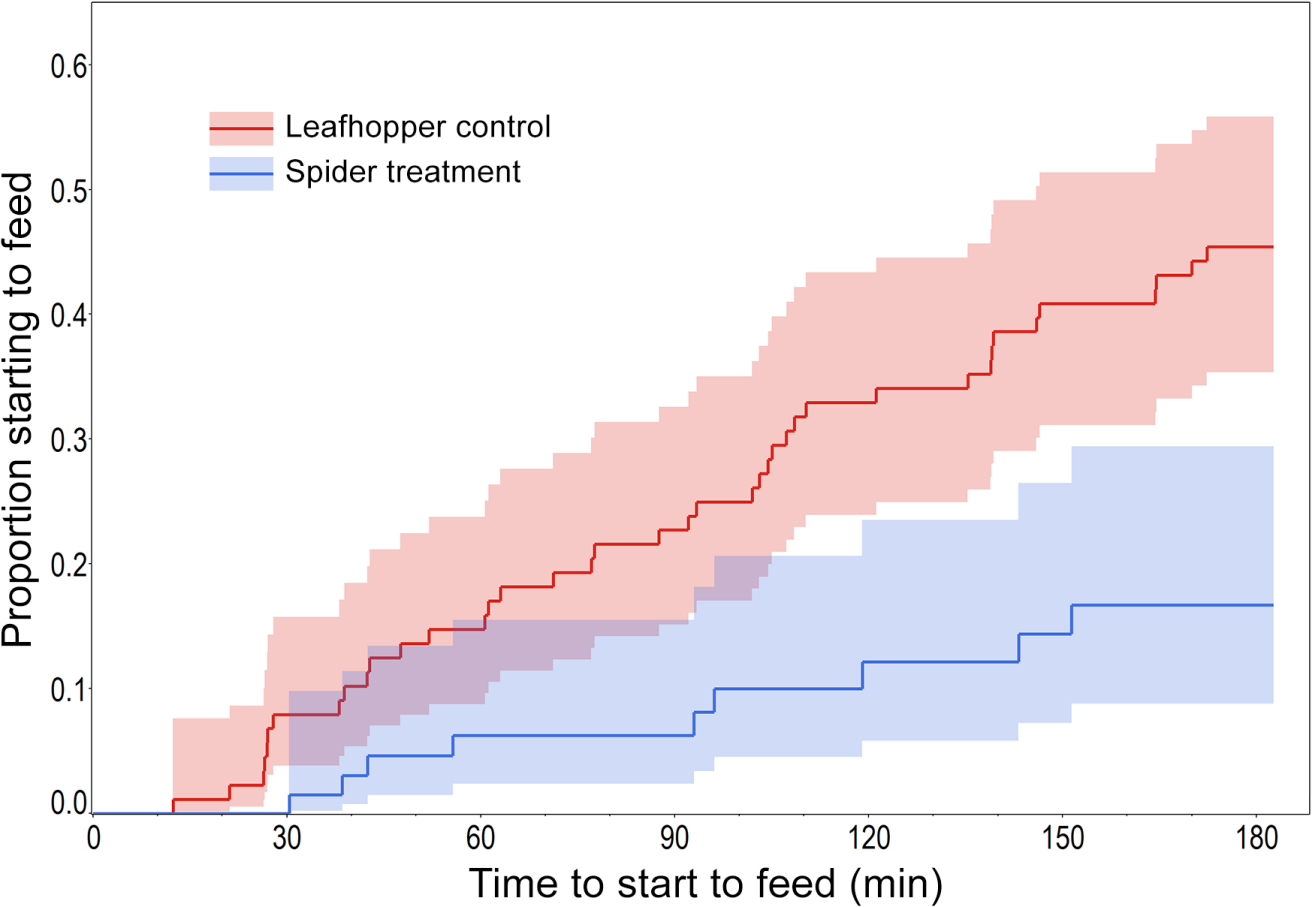
The instantaneous rate of leafhopper feeding initiation as a function of observation time. Blue line represents ‘Spider treatment’, red line ‘Leafhopper control’, shaded areas are 95% CI.

Question 3: Spider presence affected leafhopper feeding mostly by delaying it; n the spider treatment the time lag until the leafhoppers started to feed was significantly longer than in the control (Table 3 {7}, Fig 4). According to the survival analysis model, the “hazard” that a leafhopper starts to feed in the absence of spider was 3.82 times (95% CI: 1.79–10.39) greater than in the presence of spider. Male leafhoppers started feeding earlier, and the interaction between sex and spider treatment indicated that male time lag to feed was significantly less prolonged in the presence of spider than that of the females (Table 3 {7}). The duration of the feeding events was not significantly influenced by spider presence (Table 3 {8}).

In the control trials of the movement observations, which lasted only c. 30 mins, 27% of the leafhoppers started to feed, whereas in the feeding observation controls, which lasted 3 h, 45.5% of the leafhoppers started to feed. In both movement and feeding observations, in the spider treatments these figures were highly significantly lower, 3.7% and 10.2%, respectively (Table 3 {9,10}).

### Individual variation in predator-prey interactions

Leafhopper activity, including feeding, did affect the chances of becoming victim. While fewer leafhoppers fed in the spider treatments, those which did feed consistently had a lower rate of predation (Table 4), which in the feeding observations proved to be significant (Table 3 {11,12}). In those four cases when both feeding and predation occurred, the spider attacked the leafhopper after it finished feeding and started to move. No leafhopper was preyed upon while actually feeding.

**Table 4:** Number of leafhoppers started to feed, and fell as prey in the spider and control treatments during sessions of feeding and movement observations.

Feeding status	Feeding observations			Movement observations		
	Spider treatment		Control	Spider treatment		Control
	victim	Survivor		victim	survivor	
**Fed**	1	8	40	3	8	80
**Not fed**	44	35	48	132	152	216
**Total**	45	43	88	135	160	296

**Fig 5:**
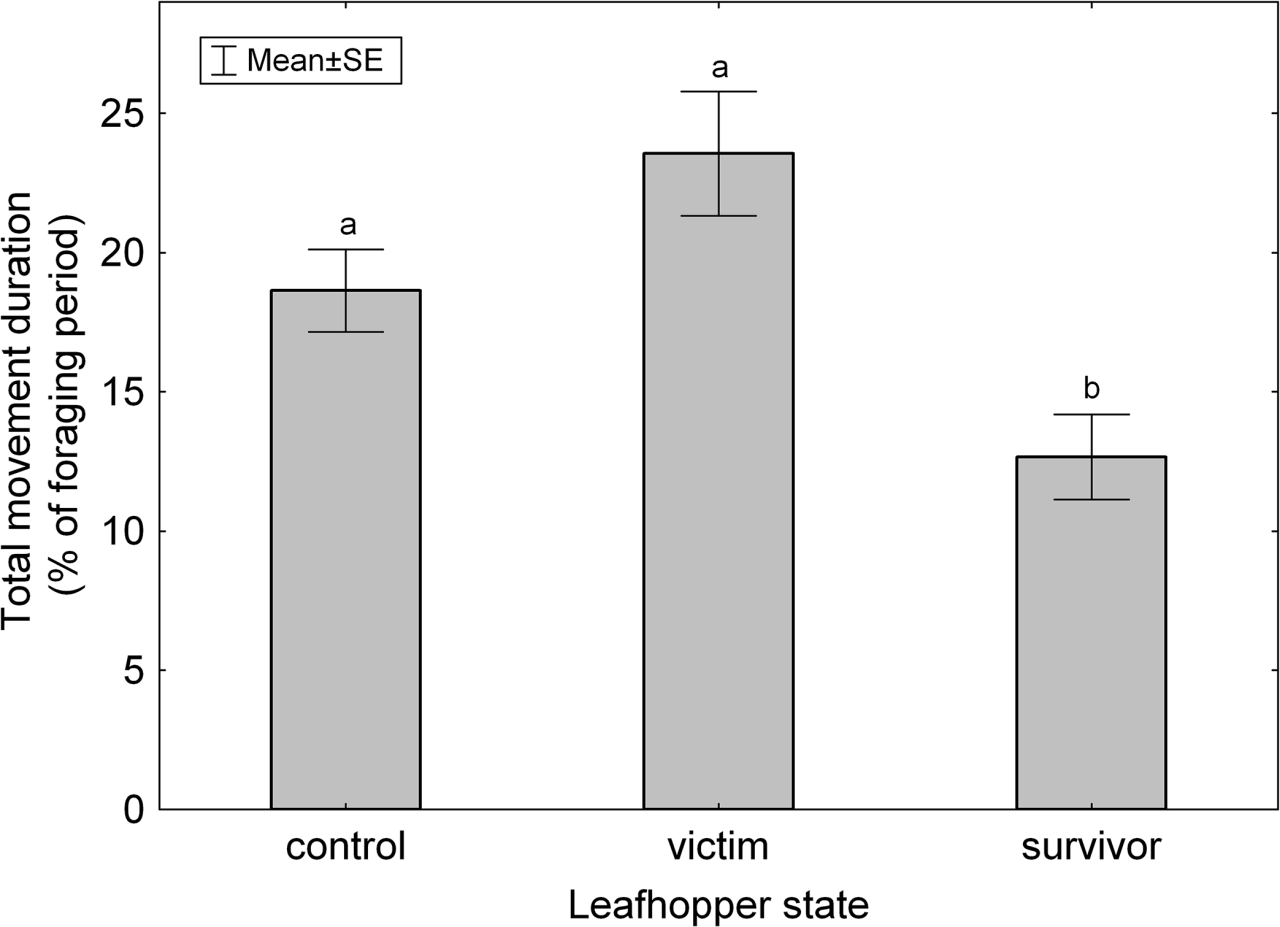
Total time spent moving expressed as percent of foraging time in control, victim and survivor leafhoppers. Groups marked with different letters are significantly different at P = 0.05 according to Tukey HSD test.

Question 4: Leafhoppers that became victims moved more frequently than survivors (Table 3 {13}), but average movement duration was not affected (Table 3 {14}). Comparing victims and survivors to control animals revealed that the percentage of time spent moving during foraging was significantly different among these groups (Fig 5, Table 3 {15}). Survivors spent less time moving than controls (*P* = 0.018), and victims tended to spend more time moving than controls (*P* = 0.051) (Fig 5).

Leafhoppers’ movement activity was not independent of the likelihood of becoming a prey (Table 3 {16}): 74.7% of the victims were moving at the moment of predation, whereas the proportion of moving individuals at the same time point was only 9.9% among the survivors.

**Fig 6:**
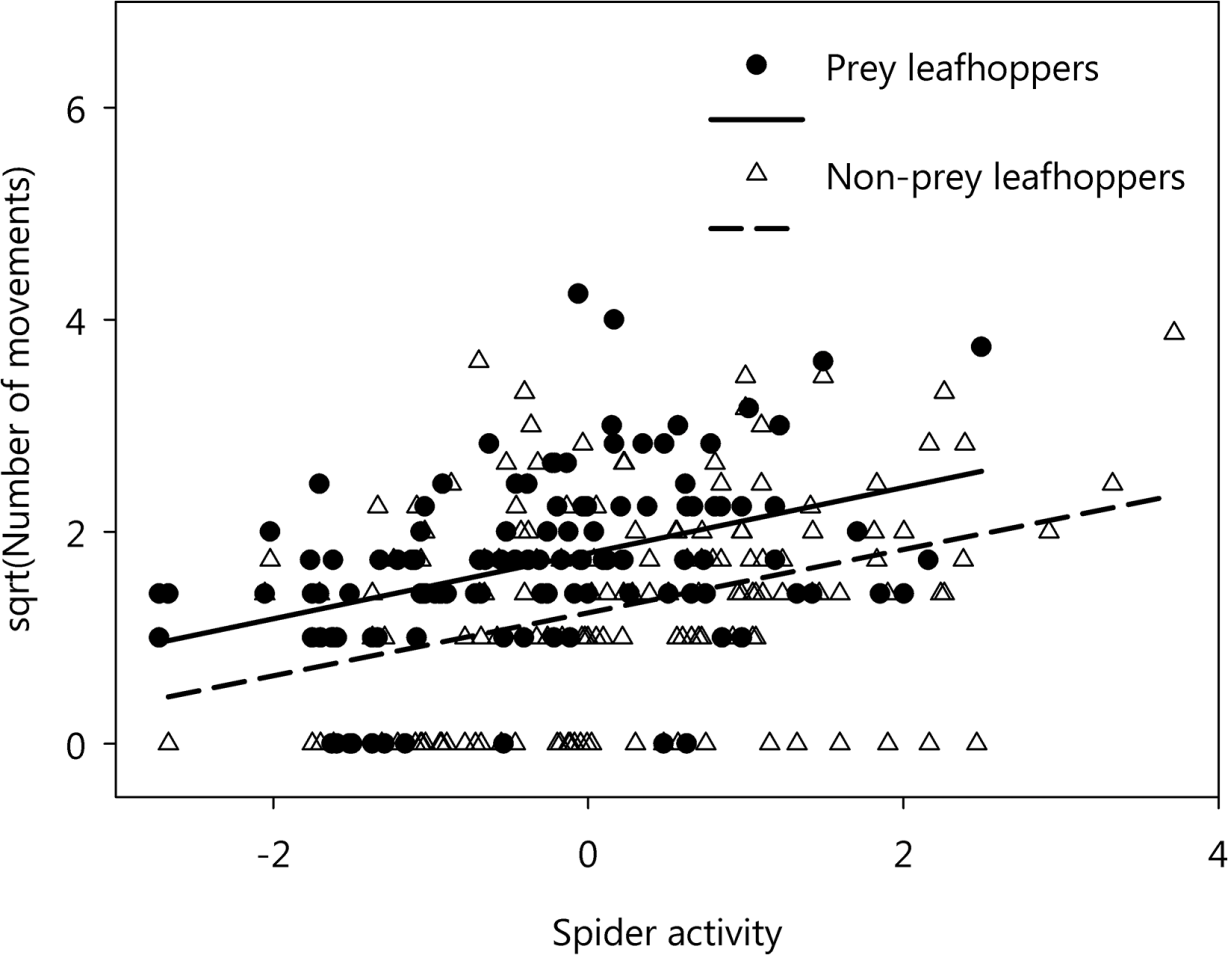
The relationship between spider activity and leafhopper movement number in prey and non-prey individuals. Spider activity was expressed as PCA first component calculated from spiders’ number and duration of movement events.

Question 5: Leafhopper movement number was significantly correlated with the spiders’ activity (Table 3 {17}). After controlling for observation duration, leafhopper movement number significantly increased if the spiders moved more (Fig 6, Table 3 {17}). The effect of the variable ‘prey’ (levels: victim/survivor) was significant in this model, as well, meaning that prospective victims and survivors differed in their movements even after accounting for spider activity (Fig 6). Surprisingly, spider size had a weak negative effect on leafhoppers’ activity. The duration of leafhoppers’ moving events was only affected by the number of movement events of spiders (Table 3 {18}).

## Discussion

The behavioural observations clearly demonstrated that leafhoppers reacted to predation risk by altering both their foraging and feeding behaviour. However, analysing movement data during foraging revealed that, independently of predator-induced stress, there is also a baseline temporal change which has to be controlled for if predation risk effects are to be detected. The baseline change manifested in both moving and stationary events becoming gradually shorter, signifying an accelerating number of movements. An acceleration of movement activity might arise from the perception of environmental gradients, e.g. chemical or tactile cues on the leaf surface that indicate the vicinity of a favourable feeding spot. Modelling studies demonstrated that even sparse sensory signals may lead the forager to concentrate search effort near feeding targets [35]. We can presume that energetic state, i.e. hunger, may also act as a driver that can be responsible for accelerated movement activity during foraging [36].

Predation risk seems to have interfered with the baseline trend by shifting the foraging towards even more frequent movements. As a result, *P*. *alienus* had more, but shorter, moving events when a spider was present in the arena. Contrary to this finding, our initial hypothesis predicted reduced activity, which was found to be a common type of anti-predator behaviour ranging from less movement [12] to death feigning [37]. However, increased movement is not necessarily dangerous for prey [13] and it can be expected if, for instance, the detection of a motionless prey increases with time [38], or if prey waste cues accumulate increasing the probability of detection over time [39]. Besides, predator presence could have interacted with physiological stress responses in the prey, as reviewed by Hawlena and Schmitz [40]. In particular, internal stress from starvation has been shown to alter anti-predator behaviour in another hemipteran, the aphid *Acyrthosiphon pisum* (Harris) [41].

Sensing killed congeners, possibly through alarm cues, are known to play a role in the detection of the predator and modulating anti-predator reaction in aphids [42]. However, the experiments reported here gave no indication of the latter mechanism in *P*. *alienus*, since leafhoppers gave the same reaction to spiders, whether they were present in the arenas or had already captured a conspecific.

Movement activity during the foraging phase was decisive for the fate of leafhopper individuals. There was a dynamic relationship between leafhopper and spider movements. Leafhoppers gave a gradated response to spiders, as they moved the least in the control trials, and when a spider was present their activity correlated with the spider’s activity. However, even after controlling for spider activity, victims still showed significantly more movements. The same phenomenon was revealed by directly comparing victims and survivors facing the same spider in the same trial. Leafhoppers are stationary while feeding, and those leafhoppers which engaged in feeding in spite of spider presence had a lower probability of predation, and in particular, no leafhopper actually feeding was preyed upon by spider. Thus, paradoxically, those individuals which showed the “typical response” of increased movement frequency to predator fell victims, and those which were”cool headed” and gave a muted response often survived.

So, why did certain individuals increase time spent moving, while others decreased it in the presence of a spider? The imperfect behaviour of victims under predation threat could be one explanation. As Welton et al. [43] argue, this might arise because there is often no second chance in learning about predation risk. Possibly, the observed behaviours might be adaptive outside the laboratory, e.g. under different predator density [44, 45]. Furthermore, our observed reaction may be generic, representing an adaptation to multiple predator species [46] with a variety of predatory tactics [47]. Leafhoppers have various spider predators [23, 48] which employ variations of sit-and-wait strategy, but also have many actively searching insect predators, like predatory bugs [49], lacewing larvae [50] and ladybird beetles [51]. Adaptation to more active predators is also hinted by the observation, that leafhopper activity was positively correlated to spider activity. If generic reaction would be the cause for the observed imperfect behaviour, that would suggest that increased predator diversity may increase prey vulnerability through limitations in adapting to various predatory tactics.

Another possible explanation for paradoxical increased activity is the risk taken by active foraging is traded off against some benefits or avoidance of other adverse effects. Frequent feeding is a necessity for sap feeding insects. They have a low endurance of starvation, because without access to sap fluids they are prone to dehydration [52]. Studies have found that starvation elicited a gradated increase in foraging movements; for instance foraging flight in the case of *Aphis fabae* (Scopoli) [53]. Poorer body condition resulting from access only to inferior host plants led the monophagous aphid *Uroleucon jaceae* (Linnaeus) to continue feeding and thus exhibit a more risk prone strategy of reduced escape behaviour in the presence of predators [54]. The time scale that starvation changes sap feeders’ behaviour is as short as 1.5 hours, during which significant changes in foraging behaviour were already detected [55]. These examples highlight the possible trade-off between condition and predation risk avoidance, so we propose that a likely mechanism for the paradoxical increased movement response of *P*. *alienus* lies in the interaction between predation risk and prey condition.

Our basic prediction that predation risk would negatively affect feeding was partly supported, as leafhoppers considerably delayed their feeding and fed much less often in the presence of a spider, although duration of feeding was not affected. Sacrificing food for safety seems to be the most common NCE of predators [8, 56], ranging from the rejection of flowers by bumble bees if a crab spider is present [57] to reduced feeding by elk after the re-introduction of wolves into Yellowstone National Park [58]. For sap feeders, patch exploitation needs an initial investment of mouthpart insertion and finding suitable vascular elements within the plant tissues. We argue that since insertion carries both a metabolically high cost and the risk of decreased escape opportunity, it is mostly the decision whether to start feeding that should depend on predation risk. The observed delayed feeding might fit a successful avoidance strategy against an average predator; however, individuals that were actually feeding during the observations experienced a substantially lower risk of predation, indicating that this strategy might be suboptimal when facing a specific sit-and-wait generalist predator.

The experiments reported here demonstrated that the presence of a spider triggers important changes in the foraging and feeding behaviour of a leafhopper, that is also vector of a plant virus and other plant diseases [18, 19]. When spider was present, feeding was delayed by *P*. *alienus*, resulting in significantly fewer feeding events. This may have important implications on virus transmission and epidemics. Details of whether, and how, the process of stylet insertion is modified under predation risk, remains to be investigated and could be important in the spread of plant pathogens such as the Wheat Dwarf Virus.

Our study showed that generalist predators, like the spider *T*. *oblongus*, exert significant non-consumptive effects on sap feeding insects. We observed the strongest reaction in reducing the number of feeding events of the leafhoppers by significantly delaying them, but the pattern of foraging movement activity was also affected. Studying individual variation in prey movements showed the interesting phenomenon of certain individuals not ceasing movement activity in the presence of predator, thus exposing themselves to higher predation risk. The observed generic strategy of leafhoppers to delay feeding when spider was present, also did not help avoiding predation. We argue that these phenomena might be due to a generic adaptation to a suite of natural enemies, which may prove imperfect facing a particular predator. A possible implication of non-consumptive effects on vector organisms is that they may cascade to further levels of the vector-pathogen-plant system, which we anticipate as an important area for future research.

## Supporting Information

S1 Table AIC values and the corresponding Akaike weights calculated for the LME models in Table 3. Candidate models for each dependent variable are sorted by their AIC value from the lowest to highest. Models in bold are the final models obtained during the applied model selection procedure. In most cases, final models had the lowest AIC value among the candidates. In model {4} and {15} the best model and the consecutive one cannot be differentiated according to their AIC value (i.e. ΔAIC<2). In model {6}, the model including ‘Predation period’ had lower AIC value (but ΔAIC was <2 between the two models) than our final model. In all these cases, we took uncertainty regarding our final model into account during the interpretation of the results.
(DOCX)

S2 Table Parameter estimates of the significant predictors in the final LME models. For each factor, the effects of additional levels (i.e. compared to the first level of the given predictor) are shown (the name of the level is written in brackets).
(DOCX)
