# Supplementary material for: Cool Headed Individuals Are Better Survivors: Non-Consumptive and Consumptive Effects of a Generalist Predator on a Sap Feeding Insect
Source: PLoS One. 2015 Aug 21;10(8):e0135954. doi: 10.1371/journal.pone.0135954 (PMC4546593; doi:10.1371/journal.pone.0135954)
Supplement: S1 Table — Candidate models for each dependent variable are sorted by their AIC value from the lowest to highest. Models in bold are the final models obtained during the applied model selection procedure. In most cases, final models had the lowest AIC value among the candidates. In model {4} and {15} the best model and the consecutive one cannot be differentiated according to their AIC value (i.e. ΔAIC<2). In model {6}, the model including ‘Predation period’ had lower AIC value (but ΔAIC was <2 between the two models) than our final model. In all these cases, we took uncertainty regarding our final model into account during the interpretation of the results. (DOCX) [file pone.0135954.s001.docx]

**STable 1. AIC values and the corresponding Akaike weights calculated for the LME models in Table 3. Candidate models for each dependent variable are sorted by their AIC value from the lowest to the highest. Models in bold are the final models obtained during the applied model selection procedure. In most cases, final models had the lowest AIC value among the candidates. In model [4] and [15] the best model and the consecutive one cannot be differentiated according to their AIC value (i.e. ΔAIC<2). In model [6], the model including ‘Predation period’ had lower AIC value (but ΔAIC was <2 between the two models) than our final model. In all these cases, we took uncertainty regarding our final model into account during the interpretation of the results.**

| **Model No.** | **Dependent variable** | **Predictors** | **AIC** | **ΔAIC** | **Relative likelihoods** | **Akaike weights** |
| --- | --- | --- | --- | --- | --- | --- |
| **[1]** | Duration of moving events^b^ | **Observation duration^b^ + Time^b^ + Spider × Sex** | **5940.19** | **0.00** | **1.00** | **0.87** |
|  |  | Observation duration^b^ + Time^b^ + Spider × Sex + Leafhopper number | 5944.89 | 4.70 | 0.10 | 0.08 |
|  |  | Observation duration^b^ + Time^b^ + Spider × Sex + Spider × Time^b^ | 5946.21 | 6.02 | 0.05 | 0.04 |
| **[2]** | Duration of stationary events^b^ | **Observation duration^b^ + Time^b^ + Spider + Leafhopper number** | **6809.05** | **0.00** | **1.00** | **0.86** |
|  |  | Observation duration^b^ + Time^b^ + Spider + Leafhopper number + Sex | 6813.91 | 4.86 | 0.09 | 0.08 |
|  |  | Observation duration^b^ + Time^b^ + Spider + Leafhopper number + Spider × Time^b^ | 6814.75 | 5.70 | 0.06 | 0.05 |
|  |  | Observation duration^b^ + Time^b^ + Spider + Leafhopper number + Spider × Sex | 6817.43 | 8.38 | 0.02 | 0.01 |
| **[3]** | Number of movements^c^ | **Observation duration + Spider + Leafhopper number** | **769.24** | **0.00** | **1.00** | **0.86** |
|  |  | Observation duration + Spider + Leafhopper number + Sex | 773.47 | 4.23 | 0.12 | 0.10 |
|  |  | Observation duration + Spider + Leafhopper number + Spider × Sex | 775.70 | 6.46 | 0.04 | 0.03 |
| **[4]** | Moving % foraging period^b^ | **Observation duration^b^** | **1485.74** | **0.00** | **1.00** | **0.47** |
|  |  | Observation duration^b^  + Sex | 1487.15 | 1.41 | 0.49 | 0.23 |
|  |  | Observation duration^b^ + Spider | 1487.70 | 1.96 | 0.38 | 0.18 |
|  |  | Observation duration^b^ + Leafhopper number | 1488.71 | 2.97 | 0.23 | 0.11 |
|  |  | Observation duration^b^ + Spider × Sex | 1492.31 | 6.57 | 0.04 | 0.02 |
| **[5]** | Number of movements^c^ | **Observation duration^b^ + Predation period** | **470.12** | **0.00** | **1.00** | **0.85** |
|  |  | Observation duration^b^ + Predation period + Predation | 474.70 | 4.58 | 0.10 | 0.09 |
|  |  | Observation duration^b^ + Predation period + Predation period × Predation | 476.30 | 6.18 | 0.05 | 0.04 |
|  |  | Observation duration^b^ + Predation period + Sex | 477.24 | 7.12 | 0.03 | 0.02 |
| **[6]** | Duration of moving events^b^ | Sex + Predation period | 392.05 | 0.00 | 1.00 | 0.48 |
|  |  | **Sex** | **393.34** | **1.29** | **0.52** | **0.25** |
|  |  | Sex + Observation duration^b^ | 394.88 | 2.83 | 0.24 | 0.12 |
|  |  | Sex + Predation | 395.32 | 3.27 | 0.19 | 0.09 |
|  |  | Sex + Predation period × Predation | 396.04 | 3.99 | 0.14 | 0.06 |
| **[13]** | Number of movements^c^ | **Observation duration^b^ + Prey** | **305.52** | **0.00** | **1.00** | **0.85** |
|  |  | Observation duration^b^ + Prey + Sex | 309.41 | 3.89 | 0.14 | 0.12 |
|  |  | Observation duration^b^ + Prey + Sex × Prey | 312.14 | 6.62 | 0.04 | 0.03 |
| **[14]** | Duration of moving events^b^ | **Observation duration^b^** | **283.21** | **0.00** | **1.00** | **0.69** |
|  |  | Observation duration^b^ + Sex | 286.25 | 3.04 | 0.22 | 0.15 |
|  |  | Observation duration^b^ + Prey | 286.45 | 3.24 | 0.20 | 0.14 |
|  |  | Observation duration^b^ + Sex × Prey | 289.79 | 6.58 | 0.04 | 0.03 |
| **[15]** | Moving % foraging period^b^ | **Observation duration^b^ + Status** | **1477.32** | **0.00** | **1.00** | **0.58** |
|  |  | Observation duration^b^ + Status + Sex | 1478.19 | 0.87 | 0.65 | 0.37 |
|  |  | Observation duration^b^ + Status + Sex × Status | 1482.27 | 4.95 | 0.08 | 0.05 |
| **[17]** | Number of movements^c^ | **Observation duration^b^ + Leafhopper number + Prey + Spider activity** | **646.19** | **0.00** | **1.00** | **0.87** |
|  |  | Observation duration^b^ + Leafhopper number + Prey + Spider activity + Prey × Spider activity | 651.14 | 4.95 | 0.08 | 0.07 |
|  |  | Observation duration^b^ + Leafhopper number + Prey + Spider activity + Spider weight | 651.71 | 5.52 | 0.06 | 0.06 |
| **[18]** | Duration of moving events^b^ | **Observation duration^b^ + Spider activity** | **618.46** | **0.00** | **1.00** | **0.66** |
|  |  | Observation duration^b^ + Spider activity + Leafhopper number | 621.10 | 2.64 | 0.267 | 0.18 |
|  |  | Observation duration^b^ + Spider activity + Prey | 622.52 | 4.06 | 0.13 | 0.09 |
|  |  | Observation duration^b^ + Spider activity + Prey × Spider activity | 623.31 | 4.85 | 0.088 | 0.06 |
|  |  | Observation duration^b^ + Spider activity + Spider weight | 625.84 | 7.38 | 0.02 | 0.02 |
