# Supplementary material for: Cool Headed Individuals Are Better Survivors: Non-Consumptive and Consumptive Effects of a Generalist Predator on a Sap Feeding Insect
Source: PLoS One. 2015 Aug 21;10(8):e0135954. doi: 10.1371/journal.pone.0135954 (PMC4546593; doi:10.1371/journal.pone.0135954)
Supplement: S2 Table — For each factor, the effects of additional levels (i.e. compared to the first level of the given predictor) are shown (the name of the level is written in brackets). (DOCX) [file pone.0135954.s002.docx]

**STable 2. Parameter estimates of the significant predictors in the final LME models. For each factor, the effects of additional levels (i.e. compared to the first level of the given predictor) are shown (the name of the level is written in brackets).**

| **Model No.** | **Observation^a^** | **Dependent variable** | **Predictors** | **Estimate (mean ± SE)** | **t-statistic** |
| --- | --- | --- | --- | --- | --- |
| **[1]** | M | Duration of moving events^b^ | Intercept | 2.24 ± 0.44 | 5.09 |
|  |  |  | Observation duration^b^ | 0.25 ± 0.05 | 4.73 |
|  |  |  | Time^b^ | -0.15 ± 0.03 | -5.46 |
|  |  |  | Sex(male) | -0.37 ± 0.14 | -2.67 |
|  |  |  | Spider(present) | -0.46 ± 0.09 | -4.85 |
|  |  |  | Spider(present) × Sex(male) | 0.51 ± 0.19 | 2.73 |
| **[2]** | M | Duration of stationary events^b^ | Intercept | 1.82 ± 0.62 | 2.91 |
|  |  |  | Observation duration^b^ | 0.57 ± 0.05 | 10.96 |
|  |  |  | Time^b^ | -0.19 ± 0.02 | -9.29 |
|  |  |  | Spider(present) | -0.44 ± 0.08 | -5.46 |
|  |  |  | Leafhopper number(two) | 0.25 ± 0.08 | 3.16 |
| **[3]** | M | Number of movements^c^ | Intercept | 1.13 ± 0.35 | 3.24 |
|  |  |  | Observation duration | 2.58 ± 0.16 | 15.84 |
|  |  |  | Spider(present) | 0.17 ± 0.05 | 3.14 |
|  |  |  | Leafhopper number(two) | -0.12 ± 0.05 | -2.27 |
| **[4]** | M | Moving % foraging period^b^ | Intercept | 1.57 ± 0.29 | 5.37 |
|  |  |  | Observation duration^b^ | -0.34 ± 0.06 | -5.79 |
| **[5]** | M | Number of movements^c^ | Intercept | -2.23 ± 0.48 | -4.62 |
|  |  |  | Observation duration^b^ | 0.49 ± 0.05 | 9.14 |
|  |  |  | Predation period(pre-attack) | 0.31 ± 0.13 | 2.49 |
| **[6]** | M | Duration of moving events^b^ | Intercept | 2.66 ± 0.11 | 23.65 |
|  |  |  | Sex(male) | 0.79 ± 0.22 | 3.62 |
| **[13]** | M | Number of movements^c^ | Intercept | 1.69 ± 0.29 | 5.79 |
|  |  |  | Observation duration^b^ | 0.35 ± 0.05 | 7.53 |
|  |  |  | Prey(true) | 0.61 ± 0.11 | 5.47 |
| **[14]** | M | Duration of moving events^b^ | Intercept | 3.36 ± 0.20 | 17.13 |
|  |  |  | Observation duration^b^ | 0.21 ± 0.07 | 2.88 |
| **[15]** | M | Moving % foraging period^b^ | Intercept | 1.71 ± 0.28 | 6.11 |
|  |  |  | Observation duration^b^ | -0.30 ± 0.06 | -4.78 |
|  |  |  | Status(survivor) | -0.31 ± 0.13 | -2.34 |
|  |  |  | Status(victim) | 0.09 ± 0.15 | 0.60 |
| **[17]** | M | Number of movements^c^ | Intercept | 2.03 ± 0.34 | 5.91 |
|  |  |  | Observation duration^b^ | 0.35 ± 0.05 | 6.75 |
|  |  |  | Leafhopper number(two) | -0.34 ± 0.10 | -3.24 |
|  |  |  | Prey(true) | 0.65 ± 0.10 6.29 | 6.29 |
|  |  |  | Spider activity | 0.10 ± 0.05 | 1.98 |
| **[18]** | M | Duration of moving events^b^ | Intercept | 3.29 ± 0.13 | 24.86 |
|  |  |  | Observation duration^b^ | 0.24 ± 0.07 | 3.48 |
|  |  |  | Spider activity | -0.19 ± 0.07 | -2.71 |

^a^Observations: M = Movement activity observation, F = Feeding observation

^b^log-transformed

^c^square-root transformed;
